# Relationships between Clinical and Non-Clinical Variables concerning Traumatic Dental Injuries in Deciduous Teeth Attended in a Children’s Hospital

**DOI:** 10.3390/children10071098

**Published:** 2023-06-22

**Authors:** Elvira Ferrés-Amat, Cristina Díaz-Martínez, Sira Herrera-Martínez, Neus Galofré-Kessler, Wilson Astudillo-Rozas, Oscar Aceituno-Antezana, Iván Valdivia-Gandur, Isabel Maura-Solivellas

**Affiliations:** 1 Paediatric Dentistry Service, Hospital HM Nens, HM Hospitales, 08009 Barcelona, Spain; elvira.fa@institutferresamat.com (E.F.-A.); mcristina_diaz@hotmail.com (C.D.-M.); sira.herrera@hotmail.com (S.H.-M.); neus_gk@hotmail.com (N.G.-K.); imaura@mail.hmhospitales.com (I.M.-S.); 2 Faculty of Dentistry, Universitat Internacional de Catalunya, 08195 Barcelona, Spain; 3 Biomedical Department, Faculty of Health Science, Universidad de Antofagasta, Antofagasta 1270300, Chile; wilson.astudillo@uantof.cl; 4 Master of Biomedical Science, Universidad de Antofagasta, Antofagasta 1270300, Chile; oscar.aceituno@uantof.cl; 5 Dentistry Department, Faculty of Medicine and Odontology, Universidad de Antofagasta, Antofagasta 1270300, Chile

**Keywords:** Traumatic Dental Injuries, deciduous teeth, non-clinical variables

## Abstract

This study aimed to describe Traumatic Dental Injuries (TDI) in a child population, with a discussion focused on the impact of non-clinical variables on TDI. A cross-sectional, descriptive, and relational study about TDI in deciduous dentition in a children’s hospital was performed. A total of 166 patients were included, of which 51.8% were male and 48.2% were female. Subluxation was the most observed injury (37.5%), and high-severity lesions predominated (60.2%). Regarding non-clinical variables, 89.2% of the patients attended urgent care centers within 24 h, and 43.4% within the first 3 h. Pointed objects were the leading cause of TDI (47%). Most TDIs were concentrated between the ages of 2 and 4 (53.5%). Concerning the place of TDI occurrence, the school (41.6%) was associated with faster urgent dental care attendance, and the home (37.3%) was associated with TDI occurrence in children under 2 years of age. Previous TDI experience (24.1% of patients) did not generate differences in the time interval between the TDI and arrival at the hospital, compared with children without a TDI history. While the behavior of clinical variables agrees with the literature reviewed, several non-clinical variables show wide differences. There is a need to identify the non-clinical variables that can significantly interact with phenomena specific to the study population (social, demographic, and cultural). The study of these variables can be useful in applying health policies. In the group studied, the non-clinical data reveals the need to educate parents or guardians on the importance of timely care in TDI, the long-term consequences of traumatism affecting deciduous dentition, and the implication of the maturation of the child’s motor skills in TDI.

## 1. Introduction

Injuries to the oral cavity comprise about 5% of all somatic injuries in all age groups, most of which are dental [1]. Traumatic Dental Injuries (TDI) are caused by an external impact on a tooth and its surrounding tissues [2]. TDI is a common affection in deciduous and permanent dentition, involving functional, esthetic, and psychological problems [3,4].

In general, TDI is considered a frequent affection in early childhood [5]. According to epidemiological studies from different countries, the frequency of deciduous tooth injury ranges from 4% to 30%, depending on patient selection methods, injury registration procedures, the diagnostic criteria applied, the classification of dental trauma, among others [6,7,8]. 

Dental trauma in deciduous teeth may cause pain and loss of function. It may also affect the development of permanent teeth and occlusion, resulting in physical, emotional, and behavioral problems for the children and their parents or guardians [9].

Dentoalveolar trauma can generate a wide variety of lesions, affecting both the tooth and its attachment apparatus. There is a consensus in the clinical classification of injuries caused by TDI [10]. However, there is a lack of consensus on how to present TDI studies and how to address the non-clinical factors often associated with TDI, such as how it happened, where it happened, or the time it took for the parents or guardians to bring the child to an urgent dental care center. The present study is developed considering the following hypothesis: “Analyzing the relationship between clinical and non-clinical variables associated with TDI provides relevant information about the behavior of this health problem in a specific community.” Therefore, this study aimed to describe the traumatisms that affected the deciduous dentition of children treated in an emergency room from a pediatric hospital, with a discussion focused on the impact of non-clinical variables on TDI. 

## 2. Materials and Methods

### 2.1. Design, Patients, and Inclusion/Exclusion Criteria

A cross-sectional, descriptive, and relational study of patients treated for TDI in their deciduous dentition in the emergency room at the Pediatric Dentistry Service, *Hospital HM Nens* (HM Hospitales) was performed. The study period was from January 2021 to July 2021. The study was authorized and approved by the Hospital’s Scientific Council and by the Ethics Committee (Act n.269 and Code of the Ethics Committee for Research with medicines of HM: 23.02.2160-GHM).

The following inclusion criteria were established for the study:

- Children treated urgently in the emergency room of the pediatric dentistry service due to TDI.
- TDIs limited to deciduous dentition.
- Children whose parents or guardians voluntarily collaborated by providing relevant information for the study.

In addition, the following exclusion criteria were established:

- Children with special needs.
- Motor disorder presented.

The study was carried out respecting the Helsinki declaration and considering the STROBE statement (see supplementary materials). A code identified each patient to respect data confidentiality. Six calibrated pediatric dentists attended to the patients, who filled in the data requested in the Case Report Form (CRF) according to the *Hospital HM Nens* procedures. The information reported in the CRF was verified given the documents of origin of the patient, if available (medical history, notes from doctors and nurses, etc.). Non-clinical information was obtained through an interview with the child’s parents/guardians. Parents or legal guardians whose children were in the study signed an informed consent to use the information obtained.

### 2.2. Variables Included in the Study

The non-clinical variables considered in the studied were “age”, “gender”, “place of TDI occurrence”, description of “how the TDI occurred”, the “time-TDI-attention” (time that elapsed from the TDI to the urgent dental care center), and “previous TDI experience” (previous history of TDI known to the parents/guardians). If the parent/guardian does not answer the queries made in relation to any variable (because he/she does not know or does not remember the answer), the variable was recorded as “does not know/does not answer”. The choice of non-clinical variables was determined based on the findings in the literature [4,5,6,7] while using the most effective method of collecting information from parents or guardians and taking into account that trauma in children can be particularly distressing for them. The variables “type of injury” and the “number of affected teeth” were diagnosed through clinical dental examination. 

To obtain the variable “how the TDI occurred”, the interviewers listened to the accounts of the parents/guardians to pool the data later (for statistical analysis).

For the variable “time-TDI-attention”, the parents or guardians were consulted regarding the time the TDI occurred, with the interviewer being the one who calculated the time from the traumatic event to dental care.

The variables “age” and “time-TDI-attention” were also analyzed as grouped variables. To carry out the “age” grouping, an adaptation of the taxonomy of psychomotor development in children prepared by Anita Harrow (1972) [11] was considered and it was divided into the following ranges: up to 2 years old (≤2 y); more than 2 years up to 4 years old (>2 y–≤4 y); and more than 4 years old (>4 y). On the other hand, the variable “time-TDI-attention” was grouped dichotomously given the following ranges: less than or equal to 3 h (≤3 h) and more than 3 h (>3 h).

The Andreasen classification [10] was used for the clinical examination to describe the “types of injury”. This classification was proposed by Petti et al. as a standard in their publication in 2022 [12], revised and validated by the WHO. A patient with a “simple diagnosis” was considered when the TDI caused one type of injury (regardless of the number of affected teeth). On the other hand, a “composite diagnosis” was considered when one or more teeth were classified with more than one type of injury. Additionally, for the purposes of this study, the types of injuries were grouped according to severity levels adapted from the work of Cohenca, et al. (2007) [13], who adapted Andreasen’s classification of the potential treatment and prognosis of the tooth plus its supporting tissues. Thus, concussion, uncomplicated crown fracture (enamel or enamel-dentin fracture, WHO 2022) [14], uncomplicated crown-root fracture, and subluxation were considered “low severity” injuries, while fracture complicated crown or crown-root, root fracture, luxation (all subtypes), and avulsion were considered “high severity” injuries. If the TDI involved more than one tooth, the patient was classified according to the most severe lesion observed. On the other hand, the lesions observed in the lip, gum, and other soft tissues were grouped into the variable “soft tissue injury”, and similar criteria were applied to form the variable “bone fractures”. Finally, the “number of affected teeth” was grouped according to the findings described in the results, and the dental nomenclature was considered for the naming of the groups.

### 2.3. Statistical Analysis of the Information

The Kolmogorov-Smirnov test was employed to examine whether the dataset originated from a normal distribution. The Mann-Whitney Wilconson or Kruskal-Wallis test was applied to analyze the distribution of continuous variables considering different grouping variables. To carry out the analysis of association or independence between variables, the Pearson’s Chi-square test or Fisher’s exact test complemented with the odds ratio was applied. The “does not know/does not answer” data were excluded from the statistical analysis; consequently, the number of responses may vary depending on the variable analyzed. SPSS 25 (Chicago, IL, USA) and G*Power Version 3.1.9.6, Heinrich-Heine-Universität Düsseldorf, Düsseldorf, Germany (post-hoc test) were used for statistical analysis. The level of significance considered was *p* < 0.05, and the Confidence Interval for the odds ratio was 95%.

## 3. Results

### 3.1. General Descriptive Analysis

A total of 166 patients met the inclusion/exclusion criteria for the study. The age of the patients fluctuated between 10 months and 7 years (mean: 3.1 years; SD 1.43). A total of 86 patients (51.8%) were male and between 1 and 6.7 years, and 80 (48.2%) were female and between 10 months and 7 years.

Concerning the place of TDI occurrence (164 responses), “school” (playgroup, childcare centers, kindergartens, among others) was the place with the highest incidence (41.6%), followed by “home” (37.3%), and other places classified under the denomination “recreational activities” (19.9%). Regarding “how the TDI occurred” (160 responses), the findings were classified as follows: caused by a “pointed object” (47%); “ground” (25.3%), the patient suffered TDI as a result of a fall and hit the ground; “blunt object or with another person” (16.9%), the impact that caused the TDI to the patient was with a blunt object or involved another person (e.g., a collision); and “wheeled vehicle” (7.2%), which includes a fall, crash, or collision from a wheeled recreational vehicle (bicycle, skate, etc.). The variable “time-TDI-attention” (153 responses) showed values from 30 min to 47 h (general average: 8.7 h; SD 9.1). A total of 89.2% of the patients attended an urgent dental care center within 24 h, and 43.4% attended one within the first 3 h (≤3). On the other hand, 24.1% reported “previous TDI experience” (166 responses).

**Figure 1:**
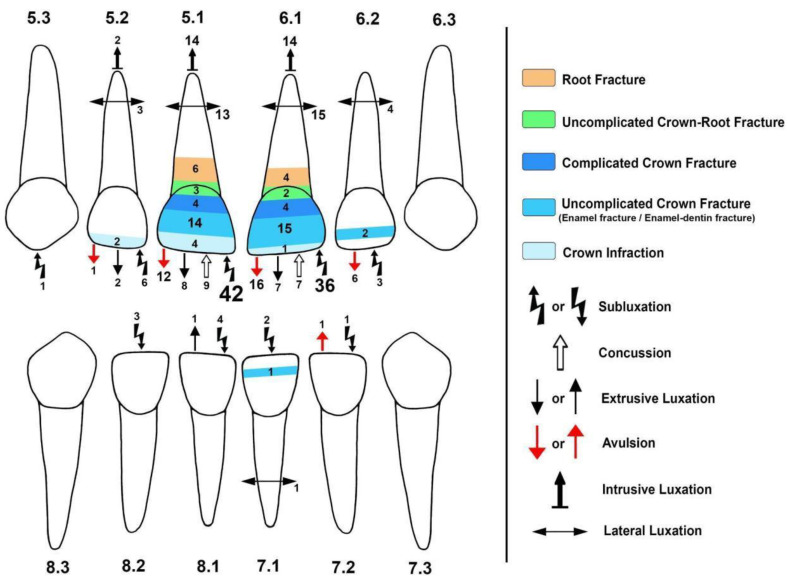
Distribution of the injuries observed using Andreasen’s classification. Note that in the WHO classification based on Andreasen’s classification (Petti et al.) [12], “uncomplicated crown fracture” encompasses “enamel fracture” and “dentin and enamel fracture.” The number close to each symbol and inside the colored areas represents the amount of each type of injury observed. The different sizes of the numbered and colored regions represent the higher or lesser concentration of a specific lesion in the tooth. The different types of injuries were mainly concentrated in teeth 5.1 and 6.1 (83.7% of the patients). Some teeth suffered more than one type of injury (30.7% of the patients).

A slight predominance of TDI was observed in male patients (51.8%). In addition, a predominance of TDI was observed between 2 and 4 years in both males (53.5%) and females (43.8%). The total number of teeth that suffered TDI in the sample was 261 teeth, 136 of which were from male (52.1%) and 125 from female (47.9%) patients. The mean number of affected teeth in boys was 1.59 (SD 0.75), while in girls it was 1.56 (SD 0.61). A total of 51.8% of the patients had TDI in one tooth, 41.6% in two teeth, 4.2% in three teeth, and 2.4% in four teeth. Considering the specific teeth injured (according to international dental nomenclature), 51 patients (30.7%) presented with a lesion in teeth 5.1–6.1, 40 patients (24.1%) with lesions only in tooth 6.1, 39 patients (23.5%) with lesions only on tooth 5.1, 9 patients (5.4%) with lesions on teeth 5.1–6.1 plus other teeth, and 27 patients (16.3%) had lesions on teeth other than 5.1 and 6.1 (Figure 1). Consequently, teeth 5.1 and 6.1 were the most affected (83.7% of patients). No TDI lesions were observed in posterior deciduous teeth. Table 1 summarizes the descriptive results. 

Concerning the “type of injury” generated by the TDI, when the analysis focused on affected teeth, subluxation was the most observed injury (98 teeth, 37.5%), followed by luxation (84 teeth, 32%, all subtypes) and avulsion (36 teeth, 13.8%); the least observed type of injury was uncomplicated crown-root fracture (5 teeth, 1.9%). However, the analysis of the type of injury grouped showed that the “high severity” lesions were mostly observed (60.2%). Additionally, 69.3% of the patients received a simple diagnosis. Figure 1 summarizes the lesions observed in the population studied by tooth, using Andreasen’s [10] classification (includes details described in the WHO 2022 classification) [12].

Regarding soft tissue and bone injuries, 90 patients (54.2%) presented with “soft tissue injuries”, while 15 patients (9%) showed “bone fractures” associated with TDI.

### 3.2. Relation between the Variables Studied

The age in the male group was significantly higher than that of the females (*p* = 0.02). The “home” as the place of TDI occurrence and the impact with “blunt object or with another person” as TDI cause, significantly affected the youngest children (*p* = 0.00 and *p* = 0.04 respectively). No significant differences in age were observed considering children grouped by severity levels of TDI (*p* = 0.60), “previous TDI experience” (*p* = 0.16), or “number of affected teeth” (*p* = 0.53).

The “number of affected teeth” was significantly higher in patients with composite diagnosis (*p* = 0.00). On the other hand, no difference was observed in the “number of affected teeth” among children grouped by gender (*p* = 0.72), age ranges studied (*p* = 0.07), severity levels of TDI (*p* = 0.60), and the “place of TDI occurrence” (*p* = 0.06). 

The time-TDI-attention showed longer times in the group less than or equal to 2 years (≤2 y, *p* = 0.04). In addition, the time-TDI-attention parameter showed significantly shorter times when the TDI was at school or during recreational activities, compared to home as the place of TDI occurrence. On the other hand, there was no difference in time-TDI-attention considering children grouped by “how the TDI occurred”, gender (*p* = 0.88), and “previous trauma experience” (*p* = 0.83).

Concerning the distribution of the types of injury, subluxation was significantly higher in the group that went to the dentist after 3 h (group > 3 h; *p* = 0.04).

Regarding the association or independence of variables, a significant relationship was observed between “home” as the place of TDI occurrence and children under 2 years of age, regardless of gender. In addition, a significant association was observed between children older than 4 years and the “school” as the place of TDI occurrence (*p* = 0.00, Table 2). Considering the variable “time-TDI-attention” dichotomized, a significant association was observed between the time spent attending urgent dental care centers for more than 3 h (>3 h) and “home” as the place of occurrence of TDI. This analysis also showed an association between “school” as the place of TDI occurrence and attending the dentist in ≤3 h (*p* = 0.00, Table 3). On the other hand, no significant association was observed between “time-TDI-attention” and “previous TDI experience” (*p* = 0.30, Table 3), which implies that those children who suffered a TDI in the past were not taken to dental care in less or more time compared to children without a history of TDI. Concerning soft tissue injuries, a significant relation was observed between the presence of soft tissue injuries and being attended at urgent care centers in less than or equal to 3 h (≤3 h). Odds ratio analysis indicated that in this group of patients, those who presented with a soft tissue injury were 2.1 times more likely to have been transferred to dental care within the first 3 h than patients without soft tissue injury. As regards the severity classification proposed in this study (high severity-low severity), a significant relationship was observed between “low severity” injuries and a lower number of “bone Fractures”. In the same way, the odds ratio showed that patients with “high severity” lesions in the group studied were 4.78 times more likely to have presented with “bone fractures” (Table 4). Additionally, the “number of affected teeth” was grouped according to the findings described in the descriptive results (“5.1–6.1”; “5.1”; “6.1”; “5.1–6.1 plus others”; “Others excluding 5.1 and 6.1”) showing a significant association with the severity levels, where the low severity was associated to patients with a lesion in teeth 5.1–6.1 (Table 4).

## 4. Discussion

According to the information published by the WHO, based on the study by Petti et al. (2018) [14,15], 20% of people worldwide suffer from TDI at some point in their lives. However, the revised literature concerning TDI showed several differences in the study design and, consequently, a wide variation in the published results. These findings agree with what has been mentioned by other authors regarding this topic [16,17,18,19]. TDI is caused mainly by accidents, so their epidemiological behavior depends on multiple variables. Moreover, within the same country, the most current hospital registry of TDI may differ from that carried out in the past due to changes in the level of insurance and legislation [17]. Considering the limitations mentioned above, the results obtained here were discussed with a focus on the behavior of non-clinical variables studied in a population affected by TDI in deciduous dentition.

### 4.1. Affected Teeth and Type of Injury as a Result of TDI in Deciduous Dentition

The upper anterior teeth were the most affected, which coincides with the clinical aspects described in the literature concerning TDI in deciduous and permanent dentition [2,8,20,21,22,23,24,25,26,27]. Considering Andreassen’s classification, a TDI can produce a wide variety of lesions (even a combination of lesions in the same tooth) which depend on the force of the impact, the object involved, child’s reaction capacity, among other non-clinical phenomena [10]. For example, subluxation and luxation (including all subtypes) were the most frequently observed lesions in the sample studied, which coincides with data observed in Santa Catarina (Brazil) [22] and Ankara (Turkey) [28]. In this regard, several authors report that luxation and avulsions are frequent in young children because they have higher bone elasticity and relatively short dental roots, which favors these type of injuries [7,21,22,27,29,30]. However, our finding differs from those observed in Ireland [31] and southern Brazil [32], where the “enamel and dentin fracture” were the most frequently observed, or in Anatolia (Turkey) [33], where a higher frequency of enamel fracture was observed. Therefore, it is understandable that there are discrepancies in the description of the TDI lesions observed in the literature. Consequently, the classification of injuries according to levels of severity or indication of treatment, like the one proposed by Cohenca et al. [13], or in the study described here, could be alternatives for operationalizing the variable for comparative or relational studies.

### 4.2. TDI in Deciduous Dentition and Its Relationship with the Gender and Age of the Patients

In general, a higher prevalence of TDI was observed in males, who also presented with a higher total number of affected teeth in the sample studied. On the other hand, the distribution of affected teeth per patient did not show significant differences between males and females. Regarding TDI in deciduous dentition, the literature shows a wide diversity of results about the prevalence of trauma considering gender. In some studies, males are the most affected; in others, it is women. On the other hand, in some studies, large percentage differences between men and women affected by trauma are observed, while in others, the differences are minimal [16,21,22,23,24,25,26,28,33,34,35,36,37,38]. Apparently, multiple aspects could influence the behavior of this variable, which are poorly addressed in the literature concerning deciduous dentition.

In the sample analyzed, a higher prevalence of TDI was observed in the 2 to 4 years age group. This is consistent with what is stated in the literature, where the level of development of motor skills in children from 2 and 3 years of age has been associated with a higher risk of TDI [5,22] because, in this stage, children test their motor skills even when their physical abilities are insufficient. It is appropriate to add that an adequate balance between the child’s weight and height is required to obtain sufficient physical capacity. In fact, a significant association between TDI and being overweight has been described since this condition has a negative impact on the balance axis of the body, making it difficult for children to develop motor skills to walk [39,40]. Therefore, recording the patient’s weight is a relevant factor to consider in TDI studies in children.

### 4.3. Place of TDI Occurrence and Its Association with Other Variables

In the cases described, most of the TDIs occurred at the school, which differs from what Azami-Aghdash et al. (2015) [41] found in their systematic review in which several articles mentioned the home as the place where the highest frequency of TDI occurred [20,25]. In addition, the study showed that children were taken to urgent care in less time when the TDI occurred at school, compared to the home as the place of TDI occurrence. However, these data must be considered with caution, considering that schools frequently have guidelines or protocols to act upon in the event of accidents that require medical attention. Instead, it is more probable that a child’s parents are likely to dismiss dental trauma as an event requiring urgent dental care, especially in deciduous dentition [22,23,27]. Moreover, these results are strongly affected by the length of time children remain in school, a variable influenced by sociocultural aspects. On the other hand, TDI in children under the age of two occurred mainly at home, probably because many younger children have not attended school yet. This suggests a differentiated approach to educating parents, guardians, or caregivers on how to prevent TDI or behave appropriately if their children suffer a TDI, considering the age and the location of the child.

Teachers and school support staff require specific training to deal with childhood TDI. In this regard, the study by Lieger et al. (2009) [42] showed that teachers with prior knowledge improve their ability to answer surveys when they are invited to participate in promotion and training campaigns to deal with children’s TDI. Consequently, how they should be trained is part of the discussion. In this sense, theoretical training through educational posters and brochures has been reported [43,44]. However, the study by Kajabuka et al. (2003) [45] showed that teachers who attended seminars and practical activities made better decisions than those who only received theoretical guidelines.

### 4.4. Timely Care in TDI of Deciduous Dentition

It is assumed that children have repetitive episodes of trauma and that males tend to have a higher risk of repeated TDI [40,46,47,48], which must be considered in the choice of treatment for the deciduous tooth. However, it could be expected that the previous TDI experience in a child generates parental learning regarding timely care. Nevertheless, the study showed that previous TDI experience did not modify the time taken to go to urgent care compared with children without a TDI history (as evidenced by the variable time-TDI-attention). This finding is consistent with reports indicating that parents often do not immediately take their children to dental care after a TDI [22,23,27,49,50,51,52], and tend to wait until the children have acute symptoms of inflammation and/or esthetic problems [27]. It should be noted that, in the study described, TDI that included soft tissue injury was significantly associated with urgent care in less time (≤3 h), probably because this event is related to increased bleeding and higher emotional distress. As a general rule, the less time that elapses between the event that produced the TDI and its care, the better its prognosis [53], which should reasonably be extended to both deciduous and permanent dentition. However, the literature shows that difficulties in establishing the period of time can be considered optimal for patients with TDI to receive urgent care. Andreasen et al. (2002) [54] describe that the treatment of TDI is generally considered acute if it is treated in a few hours (without specifying a range), subacute if the treatment is applied before 24 h, and delayed if it is applied later than 24 h. Onetto et al. (1994) [55] and Diaz et al. (2010) [37] consider different moments within the first 24 h to operationalize this variable. In the study described here, 43% of the patients received urgent care in the first 3 h after the TDI, which was consistent with the dichotomization of data used in the “time-TDI-attention” variable (≤3 h/>3 h). The attention times of TDI observed in the literature vary from some hours to 7 days, and the association of this variable with the prognosis of TDI in deciduous dentition is rare [28,35,37,53,56,57,58]. Contrarily, timely care is shown to be most relevant in permanent dentition. For example, timely treatment could avoid root resorption phenomena of teeth replanted after avulsion in permanent teeth [59]. Therefore, timely care of TDI must be correctly linked to the importance of deciduous teeth in the child’s oral health, ensuring that parents or guardians obtain adequate knowledge of this subject.

The therapeutic approach for TDI in deciduous dentition focuses on examination, monitoring, and the care and conservation of the permanent tooth associated with the traumatized area [25,30,46]. In addition, there is evidence of little psychological effect related to TDI in children, measured using the Oral Health-Related Quality of Life [60]. For these reasons, restoring the function and esthetics of the deciduous anterior teeth may be secondary to evaluating the permanent tooth health involved. Thus, the consideration of the non-clinical factors concerning TDI in deciduous dentition becomes relevant because they help us to understand the phenomenon from different perspectives. Studies from other areas of oral health have exposed the impact of the non-clinical variables [61,62,63] for example, showing the significant effect exerted by specific factors from the study population (such as social, demographic, or cultural, among others) on the behavior of the oral health problems. In this context, social and economic aspects and young parents have been related to a higher incidence of TDI in children. What is more, social factors and difficulties in accessing dental care have been linked to a lack of prompt attention [21,53].

Within the study’s limitations, most of the results were compared with data described by different authors from different places with the restrictions that this implies due to the lack of consensus in the way of presenting the TDI studies in deciduous dentition. Furthermore, the study excluded certain patients with TDI who sought direct care at the pediatric dental service of the children’s hospital without first presenting themselves to the emergency room. Therefore, although the results contribute to the opinion of experts and the subject discussion, they have particular relevance in the specific geographical region in which they were carried out.

## 5. Conclusions

In the study presented, the behavior of the clinical variables was mostly consistent with the literature reviewed. Otherwise, while the behavior of some non-clinical variables agrees with the literature reviewed, others show wide differences. There is a need to identify the non-clinical variables that can significantly interact with phenomena specific to the study population (social, demographic, cultural). These variables can be useful in applying health policies. In the group studied, the non-clinical data reveals the need to educate parents or guardians on the importance of timely care in TDI, the long-term consequences of traumatism affecting deciduous dentition, and the implication of the maturation of children’s motor skills in TDI.

## Figures and Tables

**Table 1:** Descriptive data summary. SD: standard deviation; H: home; Sch: school; Ra: recreational activity; Po: pointed object; G: ground; Bo-P: Blunt object or with another person; Wv: Wheeled vehicle; ≤3 h: up to three hours; >3 h: more than three hours; m: month; y: year.

	Patients (*n*/%)	Age Range	Age (Mean/SD)	Number of Teeth Affected (Total/Mean/SD) *n* = 261	High Severity/Low Severity *n* = 166	Place of TDI Occurrence (H/Sch/Ra) *n* = 164	How TDI Occurred (Po/G/Bo-P/Wv) *n* = 160	Time-TDI- Attention (≤3 h/>3 h) *n* = 153	Previous TDI Experience (*n*/%) *n* = 166
Male	86/51.8%	1 y to 6.7 y	3.37/ 1.5	136/1.59/0.75	53/33	29/37/19	42/22/13/6	43/43	21/12.5%
Female	80/48.2%	10 m to 7 y	2.95/1.6	125/1.56/0.61	47/33	33/32/14	36/20/15/5	42/38	19/11.4%

**Table 2:** Relationship between clinical and non-clinical variables focused on gender and age of the patients. The “*n*” varies according to the data provided by the parents or guardians. ≤2 y: up to 2 years; >2 y – ≤4y: more than 2 years up to 4 years; >4 y: more than 4 years; ≤3 h: up to three hours; >3 h: more than three hours; SP: Statistical power.

Number of Affected Teeth (*n* = 166)							
	Male	Female	*p*-value	Age ≤ 2 y	Age > 2 y– ≤4 y	Age > 4 y	*p*-value
5.1–6.1	22	17	0.50 (a)	11	18	10	0.81 (a)
5.1	20	20		12	18	10	
6.1	22	29		18	26	7	
5.1–6.1 + others	6	3		3	4	2	
Other excluding 5.1 y 6.1	16	11		5	15		
**Place of TDI occurrence (*n* = 164)**							
	**Male**	**Female**	***p*-value**	**Age ≤ 2 y**	**Age > 2 y–** **≤4 y**	**Age > 4 y**	***p*-value**
School	37	32	0.76 (a)	10	36	23	0.00 * (a) SP= 0.99
Home	29	33		31	26	5	
Recreational Activities	19	14		7	19	7	
**How the TDI occurred (*n* = 160)**							
	**Male**	**Female**	***p*-value**	**Age ≤ 2 y**	**Age > 2 y–** **≤4 y**	**Age > 4 y**	***p*-value**
Ground	22	20	0.88 (a)	7	21	14	0.11 (a)
Pointed object	42	36		28	33	17	
Blunt object or with another person	13	15		9	17	2	
Wheeled vehicle	7	5		3	6	3	
**Time-TDI-Attention (*n* = 153)**							
	**Male**	**Female**	***p*-value**	**Age ≤ 2 y**	**Age > 2 y–** **≤4 y**	**Age > 4 y**	***p*-value**
≤3 h	36	36	0.41 (b)	17	36	19	0.17 (a)
>3 h	43	38		28	40	13	
**Previous TDI experience (*n* = 166)**							
	**Male**	**Female**	***p*-value**	**Age ≤ 2 y**	**Age > 2 y–** **≤4 y**	**Age > 4 y**	***p*-value**
YES	21	19	0.53 (b)	7	23	10	0.16 (a)
NO	65	61		42	58	26	

(a) Pearson’s Chi-square test; (b) Fisher’s exact test; * Significant Association.

**Table 3:** Relationship between non-clinical variables focused on “time-TDI-attention” (≤3 h/>3 h). The “*n*” varies according to the data provided by the parents or guardians. ≤3 h: up to three hours; >3 h: more than three hours; OR (CI 95%): Odds ratio (95% Confidence Interval); SP: Statistical power.

Previous TDI Experience (*n* = 153)					
	SI	NO	*p*-value	OR (CI 95%)	
≤3 h	16	56	0.57 (a)	0.77 (0.39–1.52)	
>3 h	22	59			
**Place of TDI occurrence (*n* = 151)**					
	**School**	**Home**	**Recreational Activities**	***p*-value**	
≤3 h	41	15	15	0.00 * (b) SP= 0.99	
>3 h	23	44	13		
**How the TDI occurred (*n* = 147)**					
	**Ground**	**Pointed object**	**Blunt object or with another person**	**Wheeled vehicle**	***p*-value**
≤3 h	23	34	10	1	0.10 (b)
>3 h	17	40	15	7	
**Soft tissue injuries (*n* = 153)**					
	**YES**	**NO**	***p*** **-value**	**OR (CI 95%)**	
≤3 h	46	37	0.034 * (a)	2.1 (1.15–3.84)	
>3 h	26	44	SP = 0.68		

(a) Fisher’s exact test; (b) Pearson’s Chi-square test; * Significant Association.

**Table 4:** Relationship between clinical and non-clinical variables focused on the lesion severity (high severity/low severity). The “*n*” varies according to the data provided by the parents or guardians. ≤2 y: up to 2 years; >2 y– ≤4a: more than 2 years up to 4 years; >4a: more than 4 years; ≤3 h: up to three hours; >3 h: more than three hours; OR (CI 95%): Odds ratio (95% confidence interval). SP: Statistical power.

Gender (*n* = 166)							
	Male	Female	*p*-value	OR (CI 95%)			
High severity	53	47	0.41 (a)	1.13 (0.61–2.1)			
Low severity	33	33					
Age (*n* = 166)							
	Age ≤ 2 y	Age > 2 y – ≤4y	Age > 4 y	*p*-value			
High severity	25	55	20	0.13 (b)			
Low severity	24	26	16				
Place of TDI occurrence (*n* = 164)							
	School	Home	Recreational Activities		*p*-value		
High severity	43	36	21		0.83 (b)		
Low severity	26	26	12				
How the TDI occurred (*n* = 160)							
	Ground	Pointed Object		Blunt object or with another person		Wheeled vehicle	*p*-value
High severity	24	45		23		7	0.11 (b)
Low severity	18	33		5		5	
Time-TDI-Attention (*n* = 153)							
	≤3 h	>3 h	*p*-value	OR (CI 95%)			
High severity	47	46	0.32 (a)	1.43 (0.74–2.75)			
Low severity	25	35					
Affected teeth (*n* = 166)							
	5.1	6.1	5.1,6.1	5.1,6.1 plus others		Others excluding 5.1,6.1	*p*-value
High severity	24	28	23	8		17	0.046 * (b) SP= 0.87
Low severity	15	12	28	1		10	
Soft tissue injuries (*n* = 166)							
	YES	NO	*p*-value	OR (CI 95%)			
High severity	58	42	0.14 (a)	1.47 (0.79–2.74)			
Low severity	32	34					
Bone fractures (*n* = 166)							
	YES	NO	*p*-value	OR (CI 95%)			
High severity	13	87	0.023 * (a) SP= 0.66	4.78 (1.04–21.93)			
Low severity	2	64					

(a) Fisher’s exact test; (b) Pearson’s Chi-square test; * Significant Association.

## Data Availability

The data presented in this study are available on request from the corresponding author. The data are not publicly available due to ethical restrictions.

## Supplementary Materials

The following supporting information can be downloaded at: https://www.mdpi.com/article/10.3390/children10071098/s1, STROBE Statement—Checklist of items that should be included in reports of cross-sectional studies.

## References

[1] Petersson E.E., Andersson L., Sörensen S. (1997). Traumatic oral vs non-oral injuries. Swed. Dent. J..

[2] Altun C., Cehreli Z.C., Güven G., Acikel C. (2009). Traumatic intrusion of primary teeth and its effects on the permanent successors: A clinical follow-up study. Oral. Surg. Oral. Med. Oral. Pathol. Oral. Radiol. Endod..

[3] Borges T.S., Vargas-Ferreira F., Kramer P.F., Feldens C.A. (2017). Impact of traumatic dental injuries on oral health-related quality of life of preschool children: A systematic review and meta-analysis. PLoS ONE.

[4] Özgür B., Ünverdi G.E., Güngör H.C., McTigue D.J., Casamassimo P.S. (2021). A 3-Year retrospective study of traumatic dental Injuries to the primary dentition. Dent. Traumatol..

[5] Kurt A., Guduk O.F., Erbek S.M., Baygin O., Tuzuner T. (2019). Retrospective evaluation of patients admitted to Karadeniz Technical University Pediatric Dentistry clinic due to trauma. Eur. Oral. Res..

[6] Feldens C.A., Borges T.S., Vargas-Ferreira F., Kramer P.F. (2016). Risk factors for traumatic dental injuries in the primary dentition: Concepts, interpretation, and evidence. Dent. Traumatol..

[7] Bastone E.B., Freer T.J., McNamara J.R. (2000). Epidemiology of dental trauma: A review of the literature. Aust. Dent. J..

[8] Cunha R.F., Pugliesi D.M., de Mello Vieira A.E. (2001). Oral trauma in Brazilian patients aged 0–3 years. Dent. Traumatol..

[9] Sennhenn-Kirchner S., Jacobs H.G. (2006). Traumatic injuries to the primary dentition and effects on the permanent successors—A clinical follow-up study. Dent. Traumatol..

[10] Andreasen J., Andreasen F., Andreasen J.O., Andreasen F.M. (1994). Classification, etiology and epidemiology. Textbook and Color Atlas of Traumatic.

[11] Harrow A.A. (1972). Taxonomy of the Psychomotor Domain: A Guide for Developing Behavioral Objectives.

[12] Petti S., Andreasen J.O., Glendor U., Andersson L. (2022). NAoD—The new Traumatic Dental Injury classification of the World Health Organization. Dent. Traumatol..

[13] Cohenca N., Roges R.A., Roges R. (2007). The incidence and severity of dental trauma in intercollegiate athletes. J. Am. Dent. Assoc..

[14] WHO Oral Health. https://www.who.int/news-room/fact-sheets/detail/oral-health.

[15] Petti S., Glendor U., Andersson L. (2018). World traumatic dental injury prevalence and incidence, a meta-analysis One billion living people have had traumatic dental injuries. Dent. Traumatol..

[16] Born C.D., Jackson T.H., Koroluk L.D., Divaris K. (2019). Traumatic dental injuries in preschool-age children: Prevalence and risk factors. Clin. Exp. Dent. Res..

[17] Eissa M.A., Mustafa A.M., Splieth C.H. (2021). Dental trauma characteristics in the primary dentition in Greifswald, Germany: A comparison before and after German unification. Eur. Arch. Paediatr. Dent..

[18] Glendor U. (2008). Epidemiology of traumatic dental injuries—A 12 year review of the literature. Dent. Traumatol..

[19] Andreasen J.O., Ravn J.J. (1972). Epidemiology of traumatic dental injuries to primary and permanent teeth in a Danish population sample. Int. J. Oral. Surg..

[20] Bücher K., Neumann C., Hickel R., Kühnisch J. (2013). Traumatic dental injuries at a German university clinic 2004–2008. Dent. Traumatol..

[21] Avşar A., Topaloglu B. (2009). Traumatic tooth injuries to primary teeth of children aged 0–3 years. Dent. Traumatol..

[22] Cardoso M., de Carvalho Rocha M.J. (2002). Traumatized primary teeth in children assisted at the Federal University of Santa Catarina, Brazil. Dent. Traumatol..

[23] Ekanayake L., Perera M. (2008). Pattern of traumatic dental injuries in children attending the University Dental Hospital, Sri Lanka. Dent. Traumatol..

[24] Govindarajan M., Reddy V.N., Ramalingam K., Durai K.S., Rao P.A., Prabhu A. (2012). Prevalence of traumatic dental injuries to the anterior teeth among three to thirteen-year-old school children of Tamilnadu. Contemp. Clin. Dent..

[25] Hasan A.A., Qudeimat M.A., Andersson L. (2010). Prevalence of traumatic dental injuries in preschool children in Kuwait—A screening study. Dent. Traumatol..

[26] Kramer P.F., Zembruski C., Ferreira S.H., Feldens C.A. (2003). Traumatic dental injuries in Brazilian preschool children. Dent. Traumatol..

[27] Kirzioğlu Z., Karayilmaz H., Ertürk M.S.O., Köseler S.T. (2005). Epidemiology of raumatized primary teeth in the west-Mediterranean region of Turkey. Int. Dent. J..

[28] Arikan V., Sari S., Sonmez H. (2010). The prevalence and treatment outcomes of primary tooth injuries. Eur. J. Dent..

[29] Carvalho V., Jacomo D.R., Campos V. (2010). Frequency of intrusive luxation in deciduous teeth and its effects. Dent. Traumatol..

[30] Atabek D., Alaçam A., Aydintuğ I., Konakoğlu G. (2014). A retrospective study of traumatic dental injuries. Dent. Traumatol..

[31] Norton E., O’Connell A.C. (2012). Traumatic dental injuries and their association with malocclusion in the primary dentition of Irish children. Dent. Traumatol..

[32] Wendt F.P., Torriani D.D., Assunção M.C.F., Romano A.R., Menezes M.L., da Costa C.T., Goettems M.L., Hallal P.C. (2010). Traumatic dental injuries in primary dentition: Epidemiological study among preschool children in South Brazil. Dent. Traumatol..

[33] Unal M., Oznurhan F., Kapdan A., Aksoy S., Dürer A. (2014). Traumatic dental injuries in children. Experience of a hospital in the central Anatolia region of Turkey. Eur. J. Paediatr. Dent..

[34] Sulieman A.G., Awooda E.M. (2018). Prevalence of Anterior Dental Trauma and Its Associated Factors among Preschool Children Aged 3-5 Years in Khartoum City, Sudan. Int. J. Dent..

[35] Vuletić M., Škaričić J., Batinjan G., Trampuš Z., Čuković B.I., Jurić H. (2014). A retrospective study on traumatic dental and soft-tissue injuries in preschool children in Zagreb, Croatia. Bosn. J. Basic. Med. Sci..

[36] Arheiam A.A., Elareibi I., Elatrash A., Baker S.R. (2020). Prevalence and factors associated with traumatic dental injuries among schoolchildren in war-torn Libya. Dent. Traumatol..

[37] Díaz J.A., Bustos L., Brandt A.C., Fernández B.E. (2010). Dental injuries among children and adolescents aged 1–15 years attending to public hospital in Temuco, Chile. Dent. Traumatol..

[38] Prieto-Regueiro B., Gómez-Santos G., Diéguez-Pérez M. (2021). Prevalence of traumatic injuries in deciduous dentition and associated risk factors in a Spanish children population. J. Clin. Exp. Dent..

[39] Borges T.S., Chaffee B.W., Kramer P.F., Feldens E.G., Vítolo M.R., Feldens C.A. (2017). Relationship between overweight/obesity in the first year of age and traumatic dental injuries in early childhood: Findings from a birth cohort study. Dent. Traumatol..

[40] Magno M.B., Nadelman P., Leite K.L., de Ferreira D.M., Pithon M.M., Maia L.C. (2020). Associations and risk factors for dental trauma: A systematic review of systematic reviews. Community Dent. Oral. Epidemiol..

[41] Azami-Aghdash S., Azar F.E., Azar F.P., Rezapour A., Moradi-Joo M., Moosavi A., Oskouei S.G. (2015). Prevalence, etiology, and types of dental trauma in children and adolescents: Systematic review and meta-analysis. Med. J. Islam. Repub. Iran..

[42] Lieger O., Graf C., El-Maaytah M., Von Arx T. (2009). Impact of educational posters on the lay knowledge of school teachers regarding emergency management of dental injuries. Dent. Traumatol..

[43] Ghadimi S., Seraj B., Keshavarz H., Shamshiri A.R., Abiri R. (2014). The effect of using an educational poster on elementary school health teachers’ knowledge of emergency management of traumatic dental injuries. J. Dent. Tehran.

[44] Arikan V., Sönmez H. (2012). Knowledge level of primary school teachers regarding traumatic dental injuries and their emergency management before and after receiving an informative leaflet. Dent. Traumatol..

[45] Kahabuka F.K., Van’t-Hof M., Willemsen W., Burgersdijk R. (2003). Influence of seminar and mailed guidelines on knowledge of school teachers regarding emergency treatment for dental injuries. East. Afr. Med. J..

[46] Malmgren B., Andreasen J.O., Flores M.T., Robertson A., DiAngelis A.J., Andersson L., Cavalleri G., Cohenca N., Day P., Hicks M.L. (2016). Guidelines for the Management of Traumatic Dental Injuries: 3. Injuries in the Primary Dentition. Pediatr. Dent..

[47] Reddy L.V., Bhattacharjee R., Misch E., Sokoya M., Ducic Y. (2019). Dental Injuries and Management. Facial. Plast. Surg..

[48] Magno M.B., Neves A.B., Ferreira D.M., Pithon M.M., Maia L.C. (2019). The relationship of previous dental trauma with new cases of dental trauma. A systematic review and meta-analysis. Dent. Traumatol..

[49] Flores M.T. (2002). Traumatic injuries in the primary dentition. Dent. Traumatol..

[50] Gábris K., Tarján I., Rózsa N. (2001). Dental trauma in children presenting for treatment at the Department of Dentistry for Children and Orthodontics, Budapest, 1985–1999. Dent. Traumatol..

[51] Rai S.B., Munshi A.K. (1998). Traumatic injuries to the anterior teeth among South Kanara school children—A prevalence study. J. Indian Soc. Pedod. Prev. Dent..

[52] Osuji O.O. (1996). Traumatised primary teeth in Nigerian children attending University Hospital: The consequences of delays in seeking treatment. Int. Dent. J..

[53] Kallel I., Douki N., Amaidi S., Amor F.B. (2020). The Incidence of Complications of Dental Trauma and Associated Factors: A Retrospective Study. Int. J. Dent..

[54] Andreasen J.O., Andreasen F.M., Skeie A., Hjørting-Hansen E., Schwartz O. (2002). Effect of treatment delay upon pulp and periodontal healing of traumatic dental injuries—A review article. Dent. Traumatol..

[55] Onetto J.E., Flores M.T., Garbarino M.L. (1994). Dental trauma in children and adolescents in Valparaiso, Chile. Endod. Dent. Traumatol..

[56] Fariniuk L.F., de Souza M.H., Dietzel V.P., Carneiro E., Silva U.X., Roskamp L., Cavali A.E. (2010). Evaluation of care of dentoalveolar trauma. J. Appl. Oral. Sci..

[57] Rouhani A., Movahhed T., Ghoddusi J., Mohiti Y., Banihashemi E., Akbari M. (2015). Anterior traumatic dental injuries in East Iranian school children: Prevalence and risk factors. Iran. Endod. J..

[58] Al-Malik M. (2009). Oral injuries in children attending a hospital in Saudi Arabia. J. Maxillofac. Oral. Surg..

[59] Donaldson M., Kinirons M.J. (2001). Factors affecting the time of onset of resorption in avulsed and replanted incisor teeth in children. Dent. Traumatol..

[60] Antunes L.A.A., Lemos H.M., Milani A.J., Guimarães L.S., Küchler E.C., Antunes L.S. (2020). Does traumatic dental injury impact oral health-related to quality of life of children and adolescents? Systematic review and meta-analysis. Int. J. Dent. Hyg..

[61] Baker S.R., Pankhurst C.L., Robinson P.G. (2007). Testing relationships between clinical and non-clinical variables in xerostomia: A structural equation model of oral health-related quality of life. Qual. Life Res..

[62] Medina-Solís C.E., García-Cortés J.O., Robles-Minaya J.L., Casanova-Rosado J.F., Mariel-Cárdenas J., Ruiz-Rodríguez M.D.S., Navarrete-Hernández J.J., Ávila-Burgos L., Maupomé G. (2019). Clinical and non-clinical variables associated with preventive and curative dental service utilisation: A cross-sectional study among adolescents and young adults in Central Mexico. BMJ Open.

[63] Vermaire J.H., van Loveren C., Poorterman J.H., Hoogstraten J. (2011). Non-participation in a randomized controlled trial: The effect on clinical and non-clinical variables. Caries Res..

